# Comparison of differential accessibility analysis strategies for ATAC-seq data

**DOI:** 10.1038/s41598-020-66998-4

**Published:** 2020-06-23

**Authors:** Paul Gontarz, Shuhua Fu, Xiaoyun Xing, Shaopeng Liu, Benpeng Miao, Viktoriia Bazylianska, Akhil Sharma, Pamela Madden, Kitra Cates, Andrew Yoo, Anna Moszczynska, Ting Wang, Bo Zhang

**Affiliations:** 1 Department of Developmental Biology, Center of Regenerative Medicine, Washington University School of Medicine, St. Louis, MO 63110 USA; 2 Department of Genetics, Center for Genomic Sciences and Systems Biology, Washington University School of Medicine, St. Louis, MO 63110 USA; 3 Department of Pharmaceutical Sciences, Wayne State University, Detroit, MI 48201 USA; 4 Department of Psychiatry, Washington University School of Medicine, St. Louis, MO 63110 USA

**Keywords:** Bioinformatics, Software

## Abstract

ATAC-seq is widely used to measure chromatin accessibility and identify open chromatin regions (OCRs). OCRs usually indicate active regulatory elements in the genome and are directly associated with the gene regulatory network. The identification of differential accessibility regions (DARs) between different biological conditions is critical in determining the differential activity of regulatory elements. Differential analysis of ATAC-seq shares many similarities with differential expression analysis of RNA-seq data. However, the distribution of ATAC-seq signal intensity is different from that of RNA-seq data, and higher sensitivity is required for DARs identification. Many different tools can be used to perform differential analysis of ATAC-seq data, but a comprehensive comparison and benchmarking of these methods is still lacking. Here, we used simulated datasets to systematically measure the sensitivity and specificity of six different methods. We further discussed the statistical and signal density cut-offs in the differential analysis of ATAC-seq by applying them to real data. Batch effects are very common in high-throughput sequencing experiments. We illustrated that batch-effect correction can dramatically improve sensitivity in the differential analysis of ATAC-seq data. Finally, we developed a user-friendly package, BeCorrect, to perform batch effect correction and visualization of corrected ATAC-seq signals in a genome browser.

## Introduction

Gene regulation in the mammalian genome involves different types of regulatory elements, such as promoters, enhancers, and insulators. It was estimated that there are over two million regulatory elements in the human and mouse genomes^1,2^, and these regulatory elements recruit different epigenetic modifications to regulate the expression of genes in cell type-specific and developmental stage-specific manners^3–5^. Active regulatory elements must remain in an accessible state to allow the binding of different transcription factors to activate or silence target genes. ATAC-seq (assay for transposase-accessible chromatin followed by sequencing) is a recently developed technique to measure genome-wide chromatin accessibility (or open chromatin)^6,7^. Compared with other techniques, such as DNase-seq, Mnase-seq, and FAIRE-seq, ATAC-seq experiments are relatively easier to perform across different tissues and cell types. Furthermore, ATAC-seq experiments allow ultra-low input cell numbers, even down to the single-cell level^8^. These advantages propelled ATAC-seq to be the most widely used technology to define open chromatin by many large genomics consortiums, including ENCODE^9^, TCGA^10^, PsychENCODE^11^, IHEC^12^, and TaRGET II^13^.

The peak-calling analysis used to identify open chromatin regions (OCRs) by using ATAC-seq is generally adapted from ChIP-seq data analysis. However, there are fundamental differences between ATAC-seq and ChIP-seq - most notably that ATAC-seq is performed without control or input samples. Nonetheless, peak callers, such as macs2^14^, can identify OCRs by evaluating local enrichment against the genomic background. After peak calling, the OCRs identified in multiple samples can be combined first, and then differential analysis can be performed by estimating the difference in reads under OCRs between two different groups. Methods developed for differentially expressed gene analysis, such as edgeR^15^ and DESeq2^16^, are widely used in the differential analysis of ATAC-seq data because the general assumptions in the differential analysis of ATAC-seq data are similar to those in RNA-seq analysis^15–21^: (1). Most OCRs are the same between the two conditions, and only a small portion of OCRs are significantly different and can be identified. (2). The distribution of reads under OCRs follows a certain distribution, i.e., a negative binomial distribution. However, a comprehensive comparison of these widely used tools designed for gene expression analysis is needed to evaluate their sensitivity and specificity in the differential analysis of ATAC-seq data and thereby provide users with guidelines on method choice.

In this study, we compared the performance of four widely used software packages (DESeq ^19^, DESeq2 ^16^, edgeR^15^, and limma^18^) to that of two classic statistical methods (Wilcoxon rank-sum test and Student’s t-test). We constructed a simulated dataset on the basis of the signal distribution of real ATAC-seq data and established a common set of benchmarks to evaluate the sensitivity and specificity of the six methods in different conditions. We assessed the performance of each method with different sample sizes and sequencing depths. We further compared the performance of DESeq2 and edgeR, the two most popular software programs, using real ATAC-seq data and explored the identification of DARs with different p-value and fold-change cut-offs. We further showed that batch effect correction can greatly improve the sensitivity of ATAC-seq data analysis by removing unwanted variations and can help to identify the DARs with biological meaning. Finally, we introduced our package, BeCorrect, which can correct the batch effect of ATAC-seq signal density for visualization purposes. It is available at https://github.com/Zhang-lab/BeCorrect.

## Result

### Distinct performance of six methods of differential analysis using simulated ATAC-seq data

**Figure 1:**
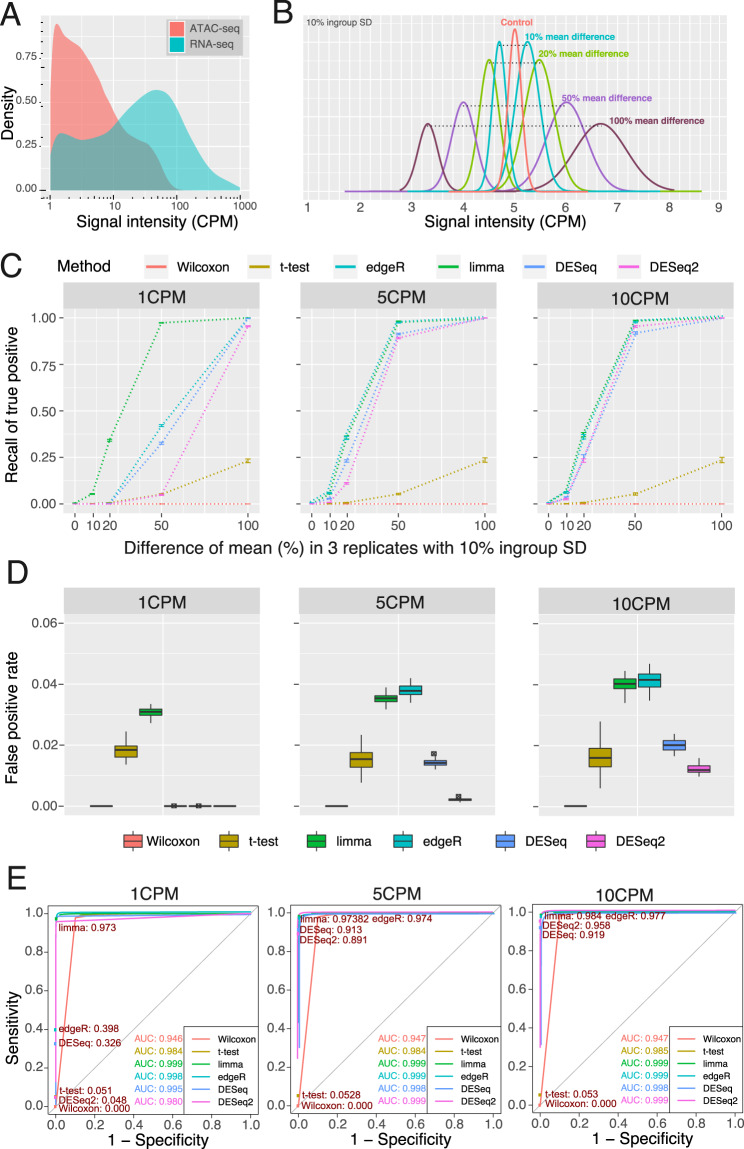
Illustration of simulation ATAC-seq data and the performance of six methods with three replicates. (**A**) Comparison of RNA-seq gene expression to ATAC-seq density. RNA-seq and ATAC-seq libraries for GM12878 were downloaded from SRA, aligned to hg38, and quantified for gene features for RNA-seq or density under peaks for ATAC-seq. Genes or peaks under 1CPM were excluded. The density of gene expression levels and peak densities was then plotted. **(B**) Simulated ATAC-seq peak density distributions. Simulated files of read counts under ATAC-seq peaks were generated as described in Methods such that they would have the desired read density under peaks. The density distribution of reads at 5 CPM with a 10% ingroup standard deviation is shown. 80% of peaks are generated as controls with equal average density in the two conditions. 5% of peaks were generated each with a 10%, 20%, 50%, or 100% mean density difference between the high density and low-density conditions. The sum of the average density of the two conditions was set as the control average density and the density difference was set such that the higher density condition had the desired density difference over the lower density condition. For example, for a 50% mean difference, the low-density condition had an average density of 4 CPM and the high density condition had an average density of 6 CPM such that their average density was 5 CPM and the high density condition was 50% more than 4 CPM. (**C**) Sensitivity of six methods with three replicates. Recall of true positives was plotted for six statistical methods as a function of increasing mean difference between high- and low-density conditions. Fifty simulated peaks files, each with over 300,000 peaks with 30 M effective reads in each file were generated as described in Methods. Average recall was plotted with error bars representing standard deviation of recall among the 50 peak files. Three replicates for the low density and high-density peaks were used in each file. **(D**) False Positive Rate of six methods with three replicates. The false positive rate was calculated for the six statistical methods by dividing the number of peaks with equal density identified as differentially accessible by the total number of differentially accessible regions identified for the 50 simulated peaks files. False positive rate was calculated separately for 1 CPM, 5 CPM, and 10 CPM using three replicates in each simulated peak file. Hinges represent the first and third quartiles. The line within the box is the median value. Whiskers extend to 1.5 times the inner quartile range. Points that are shown represent outlying points outside the whiskers. **(E**) ROC curves of six methods with three replicates. Peaks with 50% mean difference and 10% within group SD were extracted from the simulated peaks files used in (**C**) . ROC curves of sensitivity versus 1-specifity were plotted and the area under curve (AUC) calculated using the same conditions for **(D)**. Filled circles represent the sensitivity and specificity at FDR < 0.05 with the sensitivity printed for each method.

Statistical models and methods designed for gene expression analysis are widely used in the analysis of ATAC-seq data. However, unlike RNA transcripts, which can have thousands of copies per cell, the ATAC-seq signals of a given genomic region can be obtained from only two allelic DNA copies. To better understand the difference in signal distribution between RNA-seq data and ATAC-seq data, we downloaded the RNA-seq data and ATAC-seq data of GM12878^7,9^ and compared the ATAC-seq signal distribution of OCRs identified by MACS2^14^ to the RNA-seq signal distribution of expressed genes [Fig. 1A]. We noticed that over 59.4% of OCRs had low ATAC signals, from 1 counts per million (CPM) to 5 CPM, and only 23.6% of OCRs had high ATAC-seq signals, over 10 CPM. In contrast, in RNA-seq data, over 57.3% of genes had expression over 10 CPM. These left-skewed distributions of ATAC-seq signals are challenging to the sensitivity and specificity of statistical methods, especially for lower-signal OCRs, which represent a large number of distal regulatory elements, such as enhancers and insulators^1^.

In this study, we evaluated the performance of four widely used packages (DESeq, DESeq2, edgeR, and limma voom) for conducting differential analysis of chromatin accessibility. Meanwhile, we compared these four RNA-seq packages to two classic statistical tests (Wilcoxon rank-sum test and Student’s t-test) [Supplementary Table 1]. To estimate the performance of these differential analysis methods, we constructed a simulated dataset based on the signal distribution of the real data [Fig. 1B, Supplementary Fig. 1, Supplementary Table 2] (details in **Methods**). We first designed the comparison groups with 10%, 20%, 50%, and 100% mean differences in signal density at 1, 5, and 10 CPM, which represent the low, medium, and high-signal OCRs, to evaluate the sensitivity (recall of true positive) of all the methods. We further defined the control group (0% mean difference) at 1, 5, and 10 CPM to estimate the false positive rate. To standardize the comparisons, these six methods were only used to calculate the raw p-value, and Benjamini and Hochberg (BH) multiple testing corrections were performed to generate the adjusted p-value.

We first tested all six methods in the condition of three replicates [Fig. 1C], which is a typical experimental design. Both the Wilcoxon rank-sum test and the Student’s t-test had low sensitivity at any signal level. The Student’s t-test could identify ~25% of true positives when the mean difference reached 100% (2-fold change), and the Wilcoxon rank-sum test had no power to distinguish the difference at any given mean difference with three replicates. Among all the methods, limma had the highest sensitivity across all the conditions, especially in the 1 CPM group: limma identified nearly 100% of true positives with a 50% mean difference and approximately 34% of true positives with a 20% mean difference. In the 5 and 10 CPM groups, edgeR had performance comparable to limma, followed by DESeq and DESeq2.

We further calculated the false positive rate for all six methods [Fig. 1D]. Overall, all the methods can maintain a FPR < 5%, suggesting that all of these methods have good specificity, except for the Wilcoxon rank-sum test, which did not find any positive results in benchmarking with three replicates. We noticed that DESeq and DESeq2 had much better control of false positives than limma and edgeR. DESeq2 had the lowest FPR of approximately 1%, suggesting that DESeq2 had the highest specificity among all the methods. Receiver operating characteristic (ROC) curve analysis of all six methods suggested DESeq2, edgeR, and limma had very high sensitivity with low FPR when analysing high-signal groups (5CPM and 10CPM). However, both edgeR and DESeq2 showed decreased sensitivity in the low-signal group (1 CPM) [Fig. 1E]. Increasing the number of replicates to six improved the performance of the six methods when performing ROC curve analysis for equal and 50% mean difference peaks [Supplementary Fig. 2] or for the full simulated data sets [Supplementary Fig. 3].

### Sample size and sequencing depth effect on sensitivity in differential analysis

**Figure 2:**
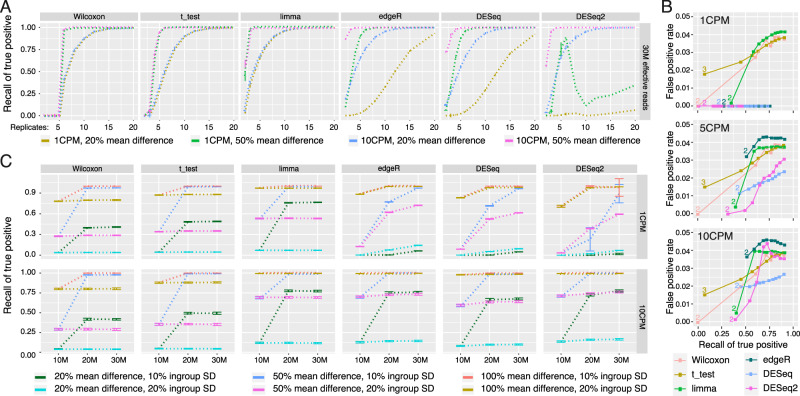
The performance of six methods with different sample size and sequencing-depth. (**A**) The effect of sample size on recall of true positives. Average recall, points, plus/minus standard deviation, error bars, across 50 simulated peak files of 30 million effective reads was calculated while varying the number of replicates used. 1 CPM and 10 CPM at 20% mean difference and 50% mean difference between higher density and lower density are shown. The ingroup variance was 10%. **(B**) ROC curve by replicates. The ROC curve of false positive rate versus recall of true positives was plotted as the number of replicates increased. True positive recall rate was calculated by summing all 10%, 20%, 50%, and 100% average density difference peaks at 1 CPM, 5 CPM, and 10 CPM. For the t-test, two replicates produced a FDR beyond the upper x limit and was not shown. For the Wilcoxon test, 2–5 replicates all did not have statistical power to call peaks as DAR. **(C**) The effect of library size and ingroup variance. Libraries with 10 M, 20 M, or 30 M effective reads were generated. Ingroup standard deviation among replicates was set at either 10% or 20% of the average read density of either 1 CPM or 10 CPM. Recall of true positives was calculated for conditions with 20%, 50%, or 100% mean difference between the high density and low-density condition for all six methods evaluated using six replicates.

Since sample size is one of the major factors that dramatically affects statistical power, we evaluated the sensitivity and specificity of the six methods as the number of replicates on each side simultaneously increased from 2 to 20 [Fig. 2A]. We noticed that the increased sample size improved the sensitivity of all the methods except for DESeq2, which had its highest sensitivity at 6 replicates of the 1 CPM group with a 50% mean difference; then, its performance dropped dramatically. DESeq2 also showed the lowest sensitivity in the 1 CPM group, with a 20% mean difference. Compared to the Wilcoxon rank-sum test, Student’s t-test, and limma, the methods based on negative binomial distribution, including edgeR, DESeq, and DESeq2, had lower sensitivity in the 1 CPM group, with a 20% mean difference. Interestingly, the Wilcoxon rank-sum test, Student’s t-test and limma showed almost 100% sensitivity in this test after the replicates reached 15. However, the Wilcoxon rank-sum test could not identify true positives with fewer than six replicates, and Student’s t-test required at least four replicates. The use of two replicates of the ATAC-seq assay per condition is a common experimental design; even the ENCODE consortium requests two replicates. With this extremely limited replicate number, limma had the best sensitivity in the 1 CPM group with a 50% mean difference; however, edgeR had the best sensitivity in the 10 CPM group with a 20% and 50% mean difference.

We further evaluated the false positive rate (FPR) of each method with increasing sample size [Fig. 2B]. All the methods showed good control of the specificity, and the overall FPR was under 5%. As the recall of true positives increased with the number of replicates, the FPR of edgeR, DESeq and DESeq2 remained at almost 0 in the 1 CPM group, with a generally lower recall of true positives. The FPR of the Wilcoxon rank-sum test, Student’s t-test and limma continued to increase with increasing replicates, as expected. With two replicates, edgeR could identify ~25% true positives of the 1 CPM group with 10%, 20%, 50%, and 100% mean difference, which was slightly lower than that of limma (~30%). However, in the 5 and 10 CPM groups, edgeR could identify ~15% more true positives with 10%, 20%, 50%, and 100% mean difference under the two replicate conditions, with a FPR ~3% higher. DESeq and DESeq2 had best controlled FPR among all the methods with a generally lower recall of true positives.

DESeq, DESeq2, and edgeR are designed based on a negative-binomial distribution; thus, the sequencing depth can directly affect the statistical power of the negative-binomial test. In our benchmarking, the Wilcoxon rank-sum test, Student’s t-test and limma used log_2_-transformed CPM as the input, and these statistical methods should not be sensitive to sequencing depth. To evaluate the influence of sequencing depth on ATAC-seq differential analysis, we simulated three different sequencing depths of 10 M, 20 M, and 30 M effective reads and tested the performance of each method in six replicate conditions [Fig. 2C]. We observed that high sequencing depth could dramatically improve the sensitivity of DESeq, DESeq2 and edgeR, especially for the 1 CPM group. We also noticed a significant improvement in sensitivity when the sequencing depth was increased from 10 M to 20 M in the Wilcoxon rank-sum test, Student’s t-test and limma test at both 20% and 50% mean difference conditions. This improvement might be caused by more accurate CPM calculation at high sequencing depth. We also performed the test at 20% within-group standard deviation (SD) to simulate a high noise situation. All the methods recalled 100% true positives in the 100% mean difference group with 20% in-group SD, and edgeR had the highest sensitivity in the 50% mean difference group with 20% in-group SD.

### Performance of DESeq2, limma, and edgeR in differential analysis of real ATAC-seq data

**Figure 3:**
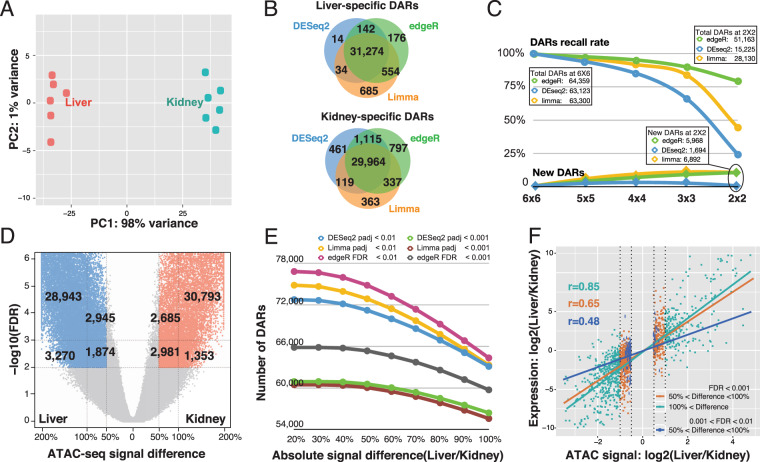
The performance of DESeq2, edgeR, and limma on real ATAC-seq data. (**A**) Principle Component Analysis of mouse liver and kidney ATAC-seq data. (**B**) Concordance of DARs identified by DESeq2, edgeR, and limma. (**C**) The effect of sample size on DARs identification by DESeq2, edgeR, and limma. X-axis indicates the sample size, Y-axis is the percentage of sensitivity (Recall rate) and false positive rate (New DARs). **(D**) Volcano-plot of DARs identified by edgeR. X-axis indicates the ATAC-seq signal difference between liver and kidney. (**E**) The effect of FDR/adjusted-p-value and signal difference on identification of DARs. X-axis is the absolute signal difference between liver and kidney. **(F**) Correlation between ATAC-seq signal and RNA-seq signal for DARs overlapping gene promoter. X-axis indicates the log_2_ transformed ATAC-seq signal difference between liver and kidney, y-axis indicates log_2_-transformed RNA-seq signal difference between liver and kidney.

DESeq,DESeq2 and edgeR are not only widely used to perform analyses for different types of omics data but are also embedded into multiple packages, such as DiffBind^22^. Our simulated ATAC-seq data suggested that DESeq/DESeq2 had better specificity and edgeR had a better sensitivity. To better evaluate the performance of different methods in real ATAC-seq differential analysis, we first downloaded published ATAC-seq data of mouse liver and kidney^23^. In this analysis, six liver samples and six kidney samples were included [Fig. 3A]. We first compared the tissue-specific DARs identified by DESeq2, edgeR, and limma with a comparable statistical cutoff (DESeq2 and limma: padj < 0.01; edgeR: FDR < 0.01). About 92.7% of total DARs can be identified by all three methods [Fig. 3B], suggesting that these three methods had very high consistency in identification of tissue-specific DARs.

We further used a down-sampling approach to evaluate the sensitivity of the three methods in this real dataset. Tissue-specific DARs were first identified in the comparison of six liver samples and six kidney samples. Then, we randomly down-sampled the replicates following DAR analysis [Fig. 3C]. In our testing, edgeR showed highest sensitivity in the condition of a low number of replicates, compared with limma and DESeq2. Even with two replicates, edegR could recall ~77% of the DARs that were identified with six replicates. Under the same conditions, DESeq2 recalled 25% of DARs and limma recalled 47% of DARs. For comparisons at each down-sampling step, we treated DARs that were newly discovered but not included in original 6 × 6 comparison condition as potential false positives. We noticed DESeq2 best controlled the potential false positive rate in all down-sampling conditions among the three methods. Meanwhile, edgeR and limma identified nearly 6,000 novel DARs in two-replicate conditions, which were considered potential false positives [Fig. 3C]. We also performed the similar test in ATAC-seq data by comparing neuronal to non-neuronal cells in human dorsolateral prefrontal cortex (DLFC)^24^. Similar to the test using mouse data, we still found DESeq2 had better control of the potential false positive, and edgeR had the highest sensitivity in all the comparisons [Supplementary Fig. 4].

In differential analysis of omics data, the q-value/FDR cut-off and fold-change cut-off were usually set empirically. FDR less than 0.01 and fold-change greater than 2 are widely used cut-offs. We studied the influence of cut-off on DAR analysis [Fig. 3D,E]. With a given FDR cut-off of 0.01 and a fold-change cut-off of over 100%, edgeR identified 32,213 liver-specific DARs and 32,146 kidney-specific DARs. We also observed an additional 4,819 liver-specific DARs and 5,666 kidney-specific DARs with FDRs less than 0.01, but the fold-change was between 50% and 100%. By reducing the fold-change cut-off from 100% to 50%, we observed a 16% increase in DARs at FDR less than 0.01 and a 9% increase in DARs at FDR less than 0.001. Interestingly, we noticed limma and DESeq2 identified fewer DARs with adjusted p-value cutoff less than 0.001 when comparing with edgeR with a FDR cutoff of less than 0.001 at all the fold-change cut-offs [Fig. 3E]. We further cross-checked the expression of corresponding genes with DARs in promoter regions. We observed a strong correlation between ATAC-seq signal changes and the expression changes of genes for the DARs with an FDR cut-off less than 0.001 and a fold-change cut-off of over 100% (r = 0.85). Interestingly, the correlation between ATAC-seq signal changes and expression changes of genes for the DARs with FDR cut-off less than 0.001 and fold-change cut-off between 50% to 100% was still as high as r = 0.65. The correlation dropped to 0.48 for the DARs with FDR cut-off between 0.001 and 0.01 and fold-change cut-off between 50% and 100% [Fig. 3F]. These results suggested that a 50% of fold-change cut-off could be favourably applied in DAR analysis when using edgeR, especially for experiments that required high sensitivity with limited replicates. For limma and DESeq2, the choice of adjusted p-value cut-off will have more impact in DAR identification.

### Removal of unwanted variables can improve the differential analysis of ATAC-seq data

Batch effects can occur at any step of an experiment, especially when some replicates are processed differently from others. In animal experiments, different cages, birth dates, and many other factors can be considered different processing conditions. They can cause large variations within groups, and eventually affect the statistical power of differential analysis between groups ^25–27^. Remove unwanted variation from RNA-Seq Data (RUVSeq) is widely used in expression analysis for both microarray platforms ^28^ and RNA-seq technology ^29^. Both edgeR and DESeq2 are also designed to account for batch effects by including batch as a covariant in their experimental design. In edgeR, this is accomplished by fitting a negative binomial generalized linear model (glm) for the batch and experimental conditions and performing the likelihood test as a generalization of the paired samples t-test. Similarly, DESeq2 fits a negative binomial glm and uses the Wald test to determine significance of the experimental condition. By contrast, RUVSeq performs factor analysis on the upper quartile normalized counts using the residuals calculated by edgeR with the end user choosing a number of unwanted factors to create normalized counts based on the removal of unwanted factors. Since RNA-seq differential analysis methods are widely applied to ATAC-seq differential analysis, we tested the performance of RUVSeq in ATAC-seq differential analysis by using real data.

**Figure 4:**
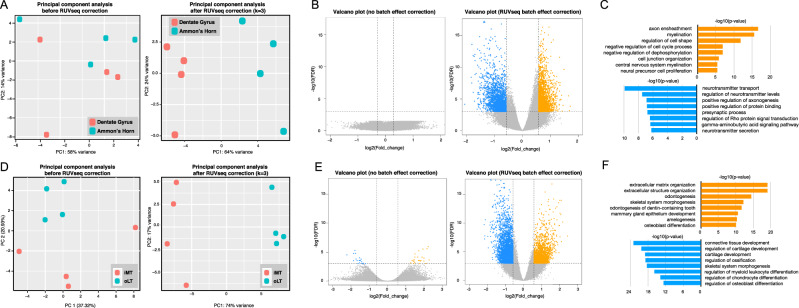
Batch-effect correction improved the performance of DAR analysis. (**A**) Principle Component Analysis of rat Dentate Gyrus and Ammon’s Horn ATAC-seq data before (left) and after (right) batch effect correction. (**B**) Volcano plots of DARs identified between Dentate Gyrus and Ammon’s Horn before (left) and after (right) batch effect correction. X-axis indicates the ATAC-seq signal difference between Dentate Gyrus and Ammon’s Horn. Y-axis indicates -log_10_(FDR). **(C**) GO enrichment analysis of genes around Dentate Gyrus (orange) and Ammon’s Horn (blue) DARs. (**D**) Principle Component Analysis of human healthy cartilage and osteoarthritis cartilage ATAC-seq data before (left) and after (right) batch effect correction. (**E**) Volcano plots of DARs identified between healthy cartilage and osteoarthritis cartilage before (left) and after (right) batch effect correction. (**F**) GO enrichment analysis of genes around osteoarthritis cartilage (orange) and human healthy cartilage (blue) DARs.

By using the omni-ATAC-seq protocol, we performed an ATAC-seq experiment to measure chromatin accessibility in the Ammon’s horn (AH, CA3) and dentate gyrus (DG, CA1) regions of the rat hippocampus. The DG is thought to contribute to the formation of new episodic memories and contain more neural stem progenitor cells. The AH is also called the hippocampus proper and contains more terminally differentiated neuronal cells. After processing the ATAC-seq data by using ATAC-seq Integrative Analysis Package (AIAP) ^30^, principal component analysis (PCA) indicated large within-group variation, and the four DG samples were mixed with the four AH samples [Fig. 4A]. After the first three unwanted variations were removed following the manual of the RUVSeq package, PCA of the corrected data showed a clear separation between DG samples and AH samples [Fig. 4A]. Based on our testing result from two real datasets, we decided to use edgeR to perform differential analysis using data corrected by edgeR and RUVSeq [Fig. 4B]. We could not identify any DAR in the data corrected by using the batch effect correction function in edgeR. However, after removing unwanted variations by RUVSeq, 2,106 DG-specific DARs and 2,117 AH-specific DARs were identified with a stringent FDR cut-off of 0.001 and a fold-change cut-off of 50% [Fig. 4B]. To understand the potential function of genes associated with these DARs, we performed GO enrichment analysis and found that genes near DARs were highly enriched in neurotransmitter transport, and positive regulation of axonogenesis [Fig. 4C]. All these biological processes were highly relevant to the functionality of the dentate gyrus and Ammon’s horn. The genes around AH-specific DARs were enriched in axon ensheathment and myelination, meanwhile, genes with negatively regulation of cell cycles were also enriched^.^ [Fig. 4D]. Snap25, a soluble N-ethylmaleimide-sensitive factor attachment protein receptor protein, plays an important role in regulating synaptic vesicle exocytosis. A DG-specific DAR can be identified at the promoter of Snap25 after the batch effect correction [Supplementary Fig. 5], suggesting the dentate gyrus-specific activation of Snap25, which confirms previous studies ^31,32^.

We further used another published ATAC-seq data of human healthy cartilage and Osteoarthritis cartilage ^33^. After removal of three unwanted variations by using RUVSeq, we could observe significant improvement in the PCA plot [Fig. 4D]. We only identified 44 DARs by using raw data before correction, and 5,056 DARs can be identified after removal of three unwanted variations by RUVSeq [Fig. 4E]. Genes around DARs were highly enriched in extracellular matrix organization, skeletal system morphogenesis, connective tissue development, cartilage development, and ossification [Fig. 4F**]**. All these results suggested that batch effect correction by removing unwanted variation could greatly improve the sensitivity of ATAC-seq differential analysis.

**Figure 5:**
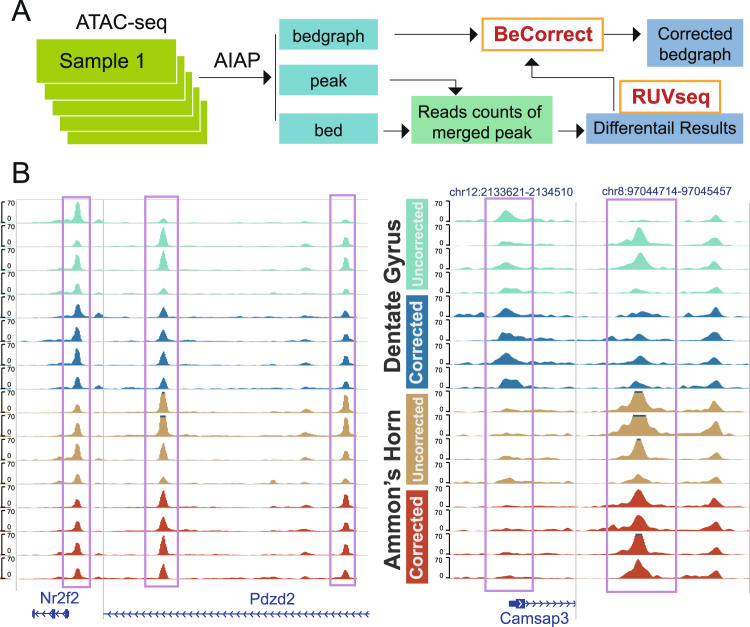
Batch-effect correction of visualized ATAC-seq signal by BeCorrect. (**A**) Flowchart for correcting ATAC-seq visualization by BeCorrect. (**B**) Genome browser view of ATAC-seq signal density before (cyan and brown) and after (blue and red) BeCorrect processing. Left: OCRs around hippocampus marker genes Nr2f2 and Pdzd2. Right: DARs on chr12 and chr8.

Most epigenomic data, including ATAC-seq, can be usually visualized on a genome browser ^34^ for a deeper exploration of the correlation to target genes or other epigenomic data. Although remove unwanted variation (RUV) can efficiently correct batch effects in the differential analysis of ATAC-seq data, the signal of ATAC-seq data (usually in bedgraph file format) cannot be directly processed by the RUVseq package. To visualize the corrected ATAC-seq signal data in the genome browser after removing the batch effects, we specifically developed a package called **Be**dgraph **Correct**or (**BeCorrect**) [Fig. 5A]. BeCorrect uses bedgraph as input, and after reading the raw read counts table and corrected read counts table, which is generated using the RUVseq package, BeCorrect calculates the corrected weights across the genome [**Methods**, Supplementary Fig. 6]. By applying BeCorrect to the ATAC-seq data of rat Ammon’s horn (AH) and dentate gyrus (DG), we could clearly observe that the corrected ATAC-seq signal was consistent with DAR analysis results, and strong batch effects were properly removed [Fig. 5B]. BeCorrect can also be used to correct the within-group variation caused by different sequencing depths, which can be considered a technical batch effect [Supplementary Figs. 5, 7].

## Discussion

ATAC-seq technology has been widely used to investigate the chromatin accessibility of regulatory elements and their functional roles in different biomedical research fields. The success and reliability of these studies depends on the accuracy of the identified differentially accessible regions (DARs). Here, we assessed the performance of six statistical methods and software packages by using both simulated and real ATAC-seq data. Our benchmarking study was designed to evaluate the performance of each method using the same dataset and statistical standard.

We first noticed that the signal distribution of ATAC-seq data was distinct from that of RNA-seq data. This difference presented certain challenges to current widely used methods that were initially designed for the differential analysis of RNA-seq data, such as DESeq/DESeq2, edgeR, and limma. Our study based on simulated data indicated that high sensitivity was important to identify DARs with low signals, especially for distal enhancers. In our test, all RNA-seq-based software had better performance than the simple Student’s t-test and Wilcoxon rank-sum test. Interestingly, limma had the highest sensitivity in most of our studies. In particular, limma had superior performance when the ATAC-seq signal was low (1 CPM), with a small mean difference (20% and 50%) between the control and treated groups in comparison. Among all the methods, DESeq/DESeq2 had the lowest false positive rate. AUC calculations for ROC analysis suggested that all the software packages tested had very strong performance for ATAC-seq signals of 5 CPM or higher. For very low ATAC-seq signal, limma had superior performance to the other methods. All these results suggested that specific methods should be chosen based on the purpose of the DAR analysis. For users who want to minimize false positive rate, we recommend using DESeq/DESeq2, whereas for users who wish to capture the highest amount of true signal, we recommend using either limma or edgeR. For users particularly concerned with detecting OCRs in low signal regions, we recommend using limma.

We also evaluated the performance of different methods as a function of different sample sizes and sequencing depths. Improved performance can be clearly observed with increased number of replicates. In our study with two replicates, we noticed that edgeR had the best sensitivity with high ATAC-seq signals (10 CPM) with a 50% mean difference, which usually indicates promoter regions. However, when the sample size reached three replicates per condition, limma achieved the highest sensitivity among all the methods. DESeq and DESeq2 showed better control of false positives than limma and edgeR; however, the overall sensitivity of these two methods was lower. In particular, DESeq2 faced difficulty when applied to DAR analysis for low-ATAC-seq-signal groups. When the sample size reached seven, the sensitivity of DESeq2 in the low-ATAC-seq-signal group (1 CPM) decreased sharply, while a high performance was maintained in the high-ATAC-seq-signal group (5 CPM and 10 CPM). This serious defect in handling low ATAC-seq signal by DESeq2 might be caused by the modified regression model of log dispersions, which is different from the previous version (DESeq)^16^. We also noticed that sequencing depth can affect the performance of differential analysis. As reported previously ^30^, the percentage of effective reads under peaks in real ATAC-seq data is determined by many factors, and is an important QC criterion to measure the quality of ATAC-seq experiments. To interpret these results from simulation testing, we suggest that three replicates should be the minimal requirement for an accurate ATAC-seq differential analysis. For each dataset, 20 M effective reads under peaks could ensure analysis with high sensitivity.

We also used real data to explore the performance of DESeq2, limma, and edgeR, the three most widely used packages for RNA-seq differential analysis. All the methods had comparable performance and high consistency with each other when six replicates were included. At decreased sample sizes, edgeR showed higher sensitivity, but with a trade-off of higher false positive rate. We further used corresponding RNA-seq data to validate the DAR results by edgeR at different fold-changes and FDR cut-offs, and we concluded that a 50% of fold-change cut-off with a stringent FDR (0.01 or less) could be recommended for DARs analysis.

We also reported that batch effect correction using the RUVSeq function RUVr can dramatically improve the sensitivity of DAR analysis when real ATAC-seq data was used and was more effective at correcting batch effects than adding batches as covariant in edgeR or DESeq2. We developed BeCorrect to generate corrected ATAC-seq signal tracks for consistent visualization in an epigenome browser. BeCorrect can be applied to other epigenomic data, including ChIP-seq and DamID-seq data. The open source code of BeCorrect is available at https://github.com/Zhang-lab/BeCorrect.

## Conclusions

To summarize our results, we marked top scoring methods based on their performance, including specificity [Figs. 1D, 2B] overall sensitivity [Fig. 2A], as well as under condition of low replicates number [Fig. 2A], high ingroup SD [Fig. 2C], and low mean difference [Fig. 1C]. We found that edgeR and limma satisfied at least two out of five criteria [Table 1]. Based on our benchmarking results using both simulated data and real data, we recommend using edgeR for the differential analysis of chromatin accessibility when high sensitivity is required or in the condition of limited sample size. Meanwhile, we recommend using DESeq2 for the DAR analysis when the best specificity is required in the condition of large sample size. To ensure sufficient sensitivity, at least three replicates per condition are recommended, and sufficient sequencing depth to improve the sensitivity of differential analysis, especially on low signal regions, such as enhancers. It is important to use PCA to check the sample distribution, and the RUV strategy can be used to correct the data to improve the sensitivity when strong batch effects are found in the data. Finally, BeCorrect can be used to correct the batch effects of the ATAC-seq data signal based on DAR analysis and generate a suitable visualization in the genome browser.

**Table 1:** Performance summary of six methods (*: top choice with best performance).

	Wilcoxon	t-test	limma	edgeR	DESeq	DESeq2
Specificity					*	*
Overall sensitivity			*	*		
Sensitivity with low replicates				*		
Sensitivity with high ingroup SD				*		
Sensitivity with low mean difference			*			

## Methods

### Comparison of RNA-seq and ATAC-seq

ATAC-seq sequence files for the GM12878 cell line for Fast-ATAC and Omni-ATAC samples were downloaded from the Sequence Read Archive (SRA). QC and downstream analysis on libraries was performed using AIAP (https://github.com/Zhang-lab/ATAC-seq_QC_analysis)^30^. Briefly, AIAP consists of four steps: data processing, quality control, integrative analysis and data visualization. **(1)** Data processing: paired-end raw reads are trimmed by cutadapt ^35^, aligned to the reference genome by bwa ^36^, and the resulting alignment BAM-format files are processed by methylQA ^36^ in the ATAC mode. **(2)** Quality control: AIAP performs multiple steps of quality checking before and after read alignment, such as reports of sequencing quality, duplication rate, GC bias by fastQC before alignment, and mapping statistics summary, chromosome distribution of uniquely mapped reads, peak width distribution after alignment. **(3)** Data visualization: It generates a collection of data visualization files that can be applied to a genome browser ^34^, including bigwig-format normalized signal density files and Tn5 insertion position files, bed-format peak files and footprint position files. It also generates JSON-format QC report files that can also be visualized via an embedded qATACviewer^30^. The generated peak files were merged using the merge function of bedtools suite, and the counts on each peak for Omni-ATAC replicate 1 were quantified using bedtools coverage. RNA-seq libraries for GM12878 were downloaded from SRA (*SRX2370562*). Raw reads were quality trimmed using cutadapt and aligned using STAR^37^, 2.5.4b to human genome (hg38 with gencode V27 GTF annotation). Alignments were quantified using featureCounts 1.6.4 ^38^, and CPM were calculated using the CPM function in edgeR^15^. Genes with expression under 1 CPM were excluded. ATAC peak counts were scaled to CPM, and peaks under 1 CPM were excluded from analysis. ATAC-seq peak density and RNA-seq expression density were plotted in R using ggplot2. Fractions of peaks and genes with expression between 1-5 CPM, 5-10 CPM, and >10 CPM were calculated.

### Construction of simulated ATAC-seq peak files with counts

To construct the simulated dataset that is similar to the real reads, we first calculated the CPM distribution within the ranges of 1-5 CPM, 5–10 CPM, and>10 CPM respectively in the mouse forebrain ENCODE ATAC-seq dataset from prenatal day 11.5 through day 16.5 and postnatal day 0 [Supplementary Fig. 1]. Based on the averaged proportion of peaks in 3 categories (1–5 CPM, 5–10 CPM, and >10 CPM) in the real data, we simulated the peak files by generating a distribution of peaks so that 60% of peaks had a signal density of 1 CPM, 30% of peaks had a density of 5 CPM, and 10% of peaks had a density of 10 CPM. For each peak, twenty replicates under each of two conditions (control vs. test) were simulated based on a gaussian distribution. At each depth, 80% of peaks were simulated such that the two conditions had equal signal density (true negatives), and 5% of peaks were simulated such that the two conditions had an average of 10%, 20%, 50%, and 100% signal density difference each (true positives). Replicates of signal density for each condition were generated by randomly selecting from a normal distribution around the mean signal density required to achieve the desired signal density difference between conditions. The standard deviation around the mean was set at either 10% or 20% of the mean for 1CPM, 5CPM, and 10CPM. For true positives, half of the simulated peaks were simulated with test condition having a higher signal density, and half of the peaks were simulated with the control having a higher signal density. The peak files meeting the conditions above were simulated at depths of 10 M, 20 M, or 30 M effective reads under peaks. For each of the three depths, a 10% or 20% ingroup standard deviation for replicates was used as previously described to generate a total of six types of peak files. For each of these six types of peaks files, we used a random seed to simulate the experiment 50 times. The C++ code to generate a simulated ATAC-seq peaks with counts file is available at (https://github.com/Zhang-lab/ATACseq_benchmarking).

### Comparisons of differential expression analysis tools

Each peak file was analysed by two statistical tests – the two-tailed Student’s t-test and the two-sided Wilcoxon rank sum test using R. For the student’s t-test, raw counts were CPM scaled and log_2_-transformed using the edgeR function “cpm” with the option ‘log = TRUE’. Additionally, each peak file was analysed by four widely used R packages for differential expression analysis (DEA) – Limma, edgeR, DESeq, and DESeq2. All comparisons were made using the default conditions for each package and function called with the single exception that the DESeq function “estimateDispersions” was called with the option fitType = “local”. Each peak file was analysed by each package or statistical comparison using all 20 replicates or by down-sampling to 15, 12, 10, 8, 6, 5, 4, 3, or 2 replicates. In all simulations, p-value adjustment was performed using the BH method with an adjusted p-value < 0.05 cut-off for significance to determine the number of each type of simulated peak called as differentially accessible unless otherwise specified. Scripts used to perform the analysis are available at https://github.com/Zhang-lab/ATACseq_benchmarking.

### FPR, TPR, recall, and ROC curves

The false positive rate (FPR) was defined according to the equation

$$FPR=\frac{FP}{FP+TN}$$

where FP, false positives, is the number of peaks called as differentially accessible from the equal mean expression levels, and TP, true positives, is the number of peaks called from those with differing mean expression levels. Specificity is defined as 1-FPR. The true positive rate (TPR), also called sensitivity, was defined according to the equation

$$TPR=\frac{TP}{FN+TP}$$

Recall was defined according to the equation

$$Recall=\frac{TP}{DAR}$$

where DAR is the total number of differentially accessible regions as defined by peaks that have differing mean expression levels. ROC curves were generated by ggplot2 in R by graphing recall against FPR using the number of replicates as the independent variable.

### Down-sampling of ATAC-seq data

ATAC-seq data of mouse liver and kidney^39^, DLFC^24^, and human cartilage^33^ were downloaded from GEO, and the QC check, data processing, and peak calling were performed by using AIAP (https://github.com/Zhang-lab/ATAC-seq_QC_analysis)^30^. Peaks identified in all samples were merged together if they had ≥1 bp overlap to generate common open chromatin regions (OCRs). The read counts of OCRs in each sample were calculated by bedtools. DARs between the liver and kidney were identified by DESeq2^16^ and edgeR^15^, respectively, with adjusted *p-values* (DESeq2) or FDR (edgeR) cut-offs of 0.01 and fold change (FC) of 100%. Down-sampling was performed by randomly excluding one replicate every time from six replicates on each side until only two replicates remained, and DAR analysis was further performed with DESeq2 or edgeR.

### Comparison of ATAC-seq and RNA-seq data

RNA-seq data of mouse liver and kidney were downloaded from the same study^39^ as the ATAC-seq data. Data were aligned to mouse genome mm10 assembly by using STAR^37^, and the expression of genes was calculated by using featureCounts from the subread package^40^. ATAC-seq peaks were further assigned to the nearest genes based on GENCODE annotation (vM15). Genes contained within tissue-specific DARs at promoter regions (2 kb regions around TSSs) were selected, and their expression fold changes were compared and calculated after TMM normalization with the edgeR package. The fold-changes of the ATAC-seq signal and RNA-seq expression were plotted in R using ggplot2 in R. GO enrichment analysis were performed by using ToppGene^41^.

### Batch correction of peak files

Library size normalization and dispersion estimate calculations on raw peak count files with multiple replicates of two conditions were performed using the R package edgeR. Removal of unwanted variations (RUV) correction was performed by following the manual of the RUVSeq package. Residuals were first calculated using RUVSeq in a general linear regression model, and the removal of unwanted variations by residuals was performed using the RUVr function to produce peak files with adjusted counts. DESeq2 and edgeR were also both used to perform batch effect correction by including batch as the first covariate in the experimental design.

### Bedgraph file adjustment

A batch-corrected peak count file and raw peak count file were used as input files to adjust the bedgraph densities based on four different criteria:

1. For bedgraph densities occurring from the start of a chromosome to the first peak in a chromosome, densities were adjusted according to the formula
  X1 $${d}_{adj}={d}_{raw}\frac{{C}_{adj}}{{C}_{raw}}$$
  where *d*_*adj*_ is the adjusted bedgraph density, d_raw_ is the original bedgraph density, *C*_*adj*_ is the adjusted peak count of the first peak in a chromosome, and C_raw_ is the raw peak count of the first peak in a chromosome.
2. Bedgraph densities occurring from the last peak in a chromosome to the end of the chromosome were adjusted according to (Eq. X1) with the modification that C_adj_ and C_raw_ represent the counts of the last peak in a chromosome from the peak count files.
3. For bedgraph densities occurring within a peak from the peak count files, densities were adjusted according to (Eq. X1) with the modification that C_adj_ and C_raw_ represent the counts of the peak where the densities are located.
4. For densities occurring between two peaks in a chromosome, densities were adjusted according to the formula

X2 $${d}_{adj}={d}_{raw}\left(\frac{({L}_{1}-{L}_{d})}{{L}_{1}-{L}_{0}}\frac{{C}_{0adj}}{{C}_{0raw}}+\frac{({L}_{d}-{L}_{0})}{{L}_{1}-{L}_{0}}\frac{{C}_{1adj}}{{C}_{1raw}}\right)$$

where L_d_ is the location of the bedgraph density, L_0_ is the location of the end of the peak before L_d_, L_1_ is the location of the start of the peak after L_d_, C_0adj_ and C_0raw_ are the adjusted and raw counts of the peak before L_d_, and C_1adj_ and C_1raw_ are the adjusted and raw counts of the peak after L_d_, respectively. BeCorrect can also accept a batch effect corrected CPM table and raw CPM table instead of count tables.

### Animal experiments

Adult male Sprague-Dawley rats (Harlan, Indianapolis, IN, USA) (weighing 250–300 g on arrival) were pair-housed under a 12 h light/dark cycle in a temperature-controlled (20–22 °C) and humidity-controlled room. Food and water were available ad libitum. The animals were allowed to acclimatize for one week before the start of the study. All animal procedures were conducted between 7:00 A.M. and 7:00 P.M. in strict accordance with the National Institutes of Health (NIH) Guide for the Care and Use of Laboratory Animals and were approved by the Institutional Animal Care and Use Committee (IACUC) at Wayne State University (animal protocol #A 16-03-067). The description of animal procedures meets the ARRIVE recommended guidelines described by The National Centre for the Replacement, Refinement and Reduction of Animals in Research ^42^.

### Brain tissue collection

Rat brains were removed and dissected out into discrete brain areas, including the dentate gyrus and Ammon’s horn. Briefly, each brain was cut coronally into 2 mm-thick slices using rat brain matrix. The dentate gyrus and Ammon’s horn (CA1 and CA3) was punched out from slices containing dorsal hippocampus at 3.6 to 5.6 mm posterior of Bregma, using fine tip 1.5 mm surgical puncher. The tissue punches were immediately frozen and stored at −80 °C until the analysis.

### ATAC-seq library construction and data processing

The Omni-ATAC protocol for nuclei isolated from frozen tissue ^7^ was slightly modified. A frozen tissue fragment of approximately 5–10 mg was placed into a pre-chilled 2 ml Dounce homogenizer containing 0.5 ml of cold 1x homogenization buffer (320 mM sucrose, 0.1 mM EDTA, 0.1% NP40, 5 mM CaCl_2_, 3 mM Mg(Ac)_2_, 10 mM Tris pH 7.8, 1x protease inhibitors(Roche, cOmplete), and 167 µM β-mercaptoethanol, in water). Tissue was homogenized with approximately 10 strokes with the larger “A” pestle, followed by 20 strokes with the narrower “B” pestle. Residual debris was removed by centrifugation for 1 minute at 100 RCF. Avoiding pelleted debris, 400 µl was transferred to a pre-chilled 2 ml round bottom Lo-Bind Eppendorf tube. An equal volume (400 µl) of a 50% iodixanol solution (50% iodixanol in 1x homogenization buffer) was added and mixed by pipetting to obtain a final concentration of 25% iodixanol. Then, 600 µl of a 29% iodixanol solution (29% iodixanol in 1x homogenization buffer containing 480 mM sucrose) was layered underneath the 25% iodixanol mixture. A clearly defined interface should be visible. In a similar fashion, 600 µl of a 35% iodixanol solution (35% iodixanol in 1x homogenization containing 480 mM sucrose) was layered underneath the 29% iodixanol solution. Again, a clearly defined interface should be visible between all three layers. In a swinging bucket centrifuge, nuclei were centrifuged for 20 minutes at 4800 RCF. After centrifugation, nuclei are present at the interface of the 29% and 35% iodixanol solutions. This band of nuclei was collected in a 300 µl volume and transferred to a prechilled tube containing 1 ml of ATAC-seq RSB (10 mM Tris pH 7.4, 10 mM NaCl, 3 mM MaCl_2_) with 0.1% Tween-20. The nuclei were pelleted by centrifugation at 500 RCF for 10 minutes in a pre-chilled 4 °C fixed-angle centrifuge. The supernatant was removed using two pipetting steps, in which the supernatant was aspirated with a p1000 pipette first, and the remaining 100 µl was carefully aspirated with a p200 pipette. Then, 20 µl of 2x TD buffer (20 mM Tris pH 7.6, 10 mM MgCl_2_, 20% dimethyl formamide) was added to the nuclear pellet and mixed by pipetting up and down 6 times. Nuclei were counted after the addition of trypan blue, which stains all nuclei. Then, 50,000 counted nuclei were transferred to a tube with 2x TD buffer filled in up to 25 µl. Then, 25 µl of transposition mix (2.5 µl transposase (100 nM final), 16.5 µl PBS, 0.5 µl 1% digitonin, 0.5 µl 10% Tween-20, 5 µl H_2_O) was added to the nuclei in 25 µl 2x TD buffer. Transposition reactions were mixed and incubated at 37 °C for 30 minutes with simple tapping every 10 minutes. Reactions were cleaned with Zymo DNA Clean and Concentrator 5 columns. The ATAC-seq library preparation was performed as described previously^6^. The ATAC-seq libraries were sequenced on an illuminated NextSeq platform with the 75 bp paired-end mode. The raw fastq of ATAC-seq data were processed by AIAP as described above, and differential analysis was performed by using edgeR. ATAC-seq peaks were further assigned to the nearest genes based on rat RefGene annotation downloaded from UCSC. GO enrichment analysis were performed by using ToppGene ^41^.

## Supplementary information

Supplementary Figures S1-7, Tables S1-2.

Supplementary Table S3.

## Data Availability

The software, source code, and documentation are freely available at https://github.com/Zhang-lab/BeCorrect and https://github.com/Zhang-lab/ATACseq_benchmarking. ATAC-seq data of rat brain tissues is available under GEO accession number GSE131144.
