# Supplementary material for: Comparison of differential accessibility analysis strategies for ATAC-seq data
Source: Sci Rep. 2020 Jun 23;10:10150. doi: 10.1038/s41598-020-66998-4 (PMC7311460; doi:10.1038/s41598-020-66998-4)
Supplement: Supplementary file 1 — Supplementary Figures S1-7, Tables S1-2. [file 41598_2020_66998_MOESM1_ESM.docx]

**Title: Comparison of differential accessibility analysis strategies for ATAC-seq data**

Paul Gontarz^1^, Shuhua Fu^1^, Xiaoyun Xing^2^, Shaopeng Liu^1^, Benpeng Miao^1^, Viktoriia Bazylianska^3^, Akhil Sharma^3^, Pamela Madden^4^, Kitra Cates^1^, Andrew Yoo^1^, Anna Moszczynska^3^, Ting Wang^2*^, Bo Zhang^1*^

1. Department of Developmental Biology, Center of Regenerative Medicine, Washington University School of Medicine, St. Louis, MO, 63108, USA
2. Department of Genetics, Center for Genomic Sciences and Systems Biology, Washington University School of Medicine, St. Louis, MO, 63108, USA
3. Department of Pharmaceutical Sciences, Wayne State University, Detroit, MI, 48201, USA
4. Department of Psychiatry, Washington University School of Medicine, St. Louis, MO, 63108, USA

* To whom correspondence should be addressed.

Tel: +1 (314)286-0865; Fax: +(314) 362-7855; Email: [twang@wustl.edu](mailto:twang@wustl.edu)

Tel: +1 (314)362-4757; Fax: +(314) 362-7051; Email: [bzhang29@wustl.edu](mailto:bzhang29@wustl.edu)

**Supplementary table 1: Description of 6 methods used in the study**

**Supplementary table 2: Details of ATAC-seq simulation data in distinct CPM**

**Supplementary table 3: Data in Fig1 and Fig2 (Supplementary_table_3.xlsx)**

**Supplementary figure 1: Distribution of ATAC-seq peak signal densities in real and simulated datasets.**

**Supplementary figure 2: ROC curves of 6 replicates with 50% mean difference for 20M and 30M effective reads.**

**Supplementary figure 3: ROC curves of 6 replicates for full simulated datasets for 20M and 30M effective reads.**

**Supplementary figure 4: The performance of DESeq2, edgeR, and limma on real ATAC-seq data**

**Supplementary figure 5: Differential accessible region between Dentate gyrus and Ammon’s horn located in the promoter of Snap25.**

**Supplementary figure 6: BeCorrect adjust the bedgraph densities based on four different criteria.**

**Supplementary figure 7: BeCorrect corrected the technical batch effect (sequencing-depth).**

**Supplementary table 1: Description of 6 methods used in the study**

| Method | Version | Statistical test | Input Data | Normalization |
| --- | --- | --- | --- | --- |
| DESeq2 | 1.18.1 | Wald test on Negative Binomial GLM | Counts table | Median Ratio Method |
| DESeq | 1.30.0 | Negative Binomial | Counts table | Size factor as geometric mean of genes |
| edgeR | 3.20.9 | Negative Binomial / Generalized Linear Model | Counts table | TMM normalization |
| limma | 3.34.9 | Empirical Bayes moderated t-statistic | log2 cpm table | log2 cpm normalization |
| student t-test | 3.5.1 | two-tailed student t-test uneuqal variance | log2 cpm table | log2 cpm normalization |
| Wilcoxon | 3.5.1 | two.sided Wilcoxon test | cpm table | log2 cpm normalization |

**Supplementary table 2: Details of ATAC-seq simulation data in distinct CPM**

|  | Condition | Peaks | Note |
| --- | --- | --- | --- |
| 1CPM | equal | 154835 | Ture negative |
|  | 10diff | 9678 | Ture positive |
|  | 20diff | 9678 | Ture positive |
|  | 50diff | 9678 | Ture positive |
|  | 100diff | 9678 | Ture positive |
| 5CPM | equal | 77418 | Ture negative |
|  | 10diff | 4838 | Ture positive |
|  | 20diff | 4838 | Ture positive |
|  | 50diff | 4838 | Ture positive |
|  | 100diff | 4838 | Ture positive |
| 10CPM | equal | 25806 | Ture negative |
|  | 10diff | 1612 | Ture positive |
|  | 20diff | 1612 | Ture positive |
|  | 50diff | 1612 | Ture positive |
|  | 100diff | 1612 | Ture positive |

**Supplementary Figure 1. Distribution of ATAC-seq peak signal densities in real and simulated datasets.** The distribution of signal densities of ATAC-seq peaks for 14 mouse forebrain samples from ENCODE samples from prenatal day 11.5 through prenatal day 16.5 and postnatal day 0 were determined. Peaks with signal density ranging from 0.5CPM to less than 2.5CPM were counted as 1CPM. Peaks with signal density ranging from 2.5CPM to less than 10 CPM were counted as 5CPM. Peaks with signal density over 10CPM were counted as 10CPM. The fraction of peaks for each sample were plotted as a boxplot with the lower and upper hinges corresponding to the first and third quartiles respectively and the upper whisker extending to 1.5 (inter quartile range). The fraction of peaks at each depth in the simulated dataset is shown as purple circles.


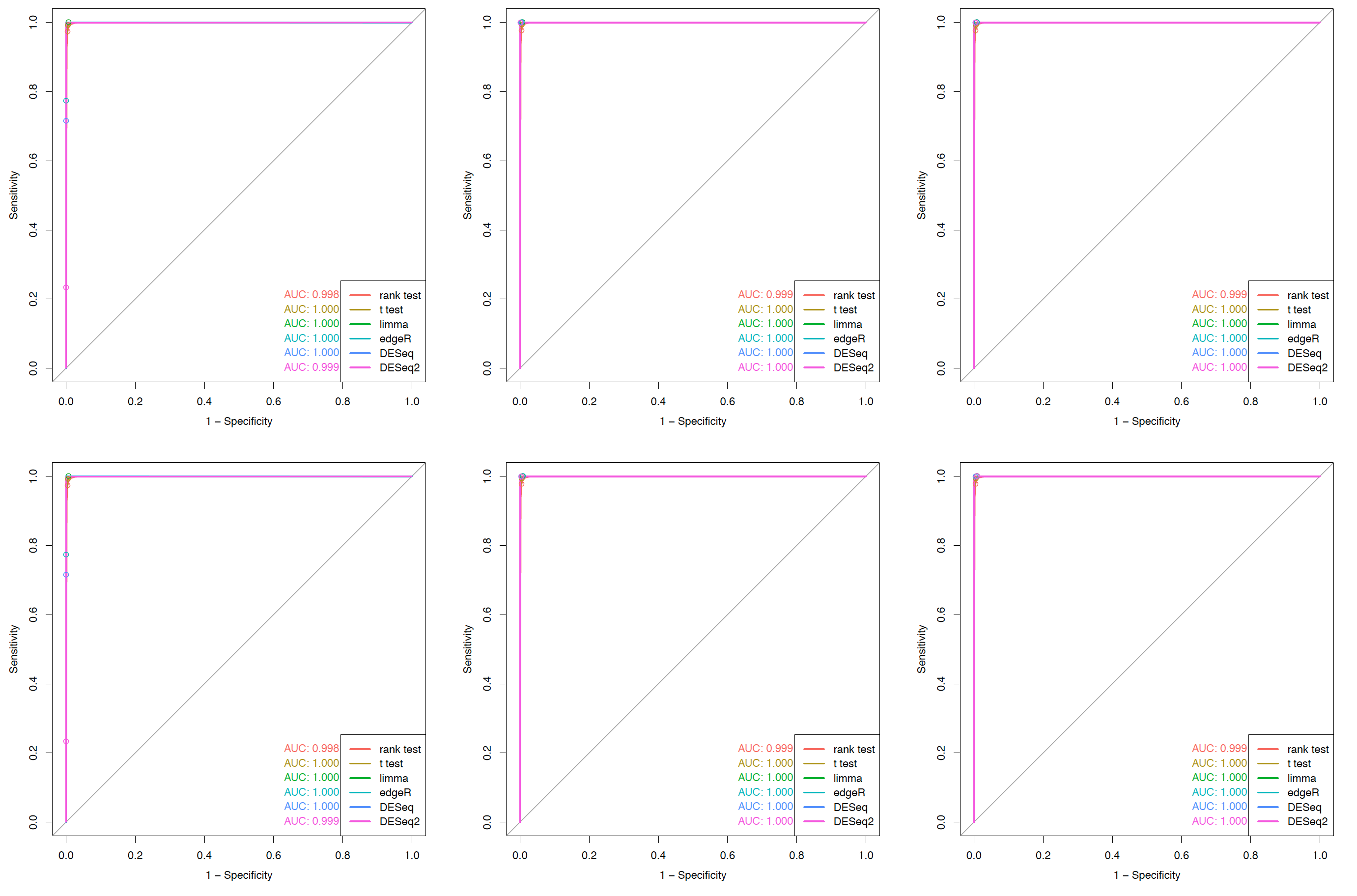


**Supplementary Figure 2.**  **ROC curves of 6 replicates with 50% mean difference for 20M and 30M effective reads.** ROC curves were plotted for simulated data using 6 replicates with equal signal density and 50% difference signal density peaks for 20M (top row) or 30M (bottom row) effective reads at 1CPM (left column), 5CPM (center column) or 10CPM (right column). Open circles represent sensitivity and selectivity at FDR<0.05.


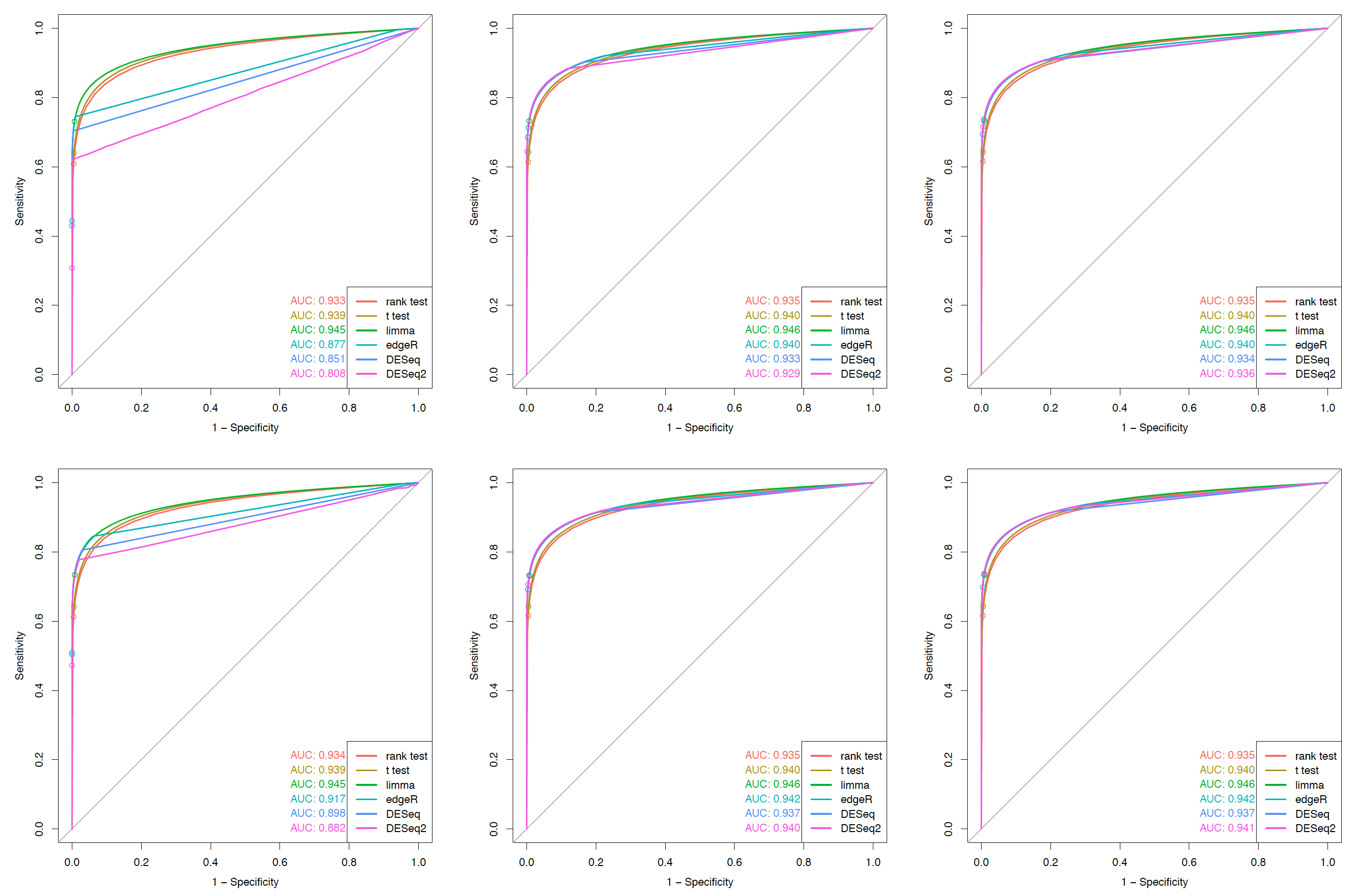


**Supplementary Figure 3.**  **ROC curves of 6 replicates for full simulated datasets for 20M and 30M effective reads**. ROC curves were plotted for simulated data using 6 replicates with equal signal density and 10%, 20%, 50%, and 100% difference signal density peaks for 20M (top row) or 30M (bottom row) effective reads at 1CPM (left column), 5CPM (center column) or 10CPM (right column). Open circles represent sensitivity and selectivity at FDR<0.05.


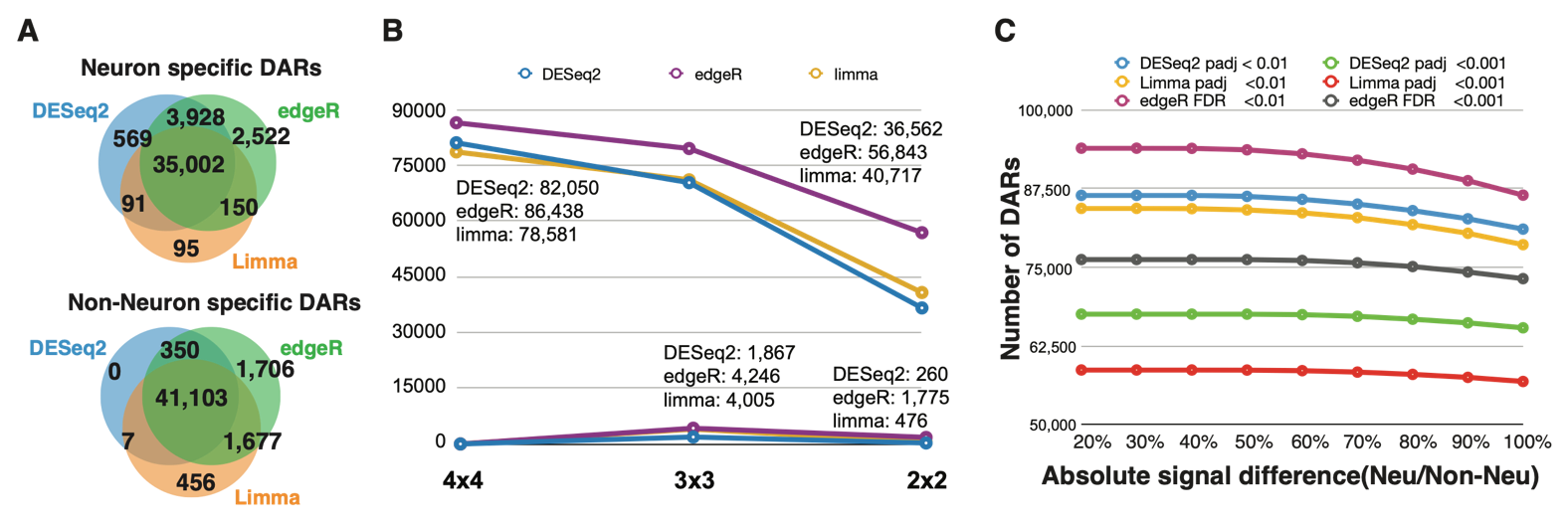


**Supplementary Figure 4.** **The performance of DESeq2, edgeR, and limma in neuron and non-neuron data.** **A). Concordance of DARs identified by DESeq2, edgeR, and limma. B). The effect of sample size on DARs identification by DESeq2, edgeR, and limma.** X-axis indicates the sample size, Y-axis is the number recalls and false positive rate (New DARs). **C). The effect of FDR/adjusted-p-value and signal difference on identification of DARs.** X-axis is the absolute signal difference between liver and kidney.


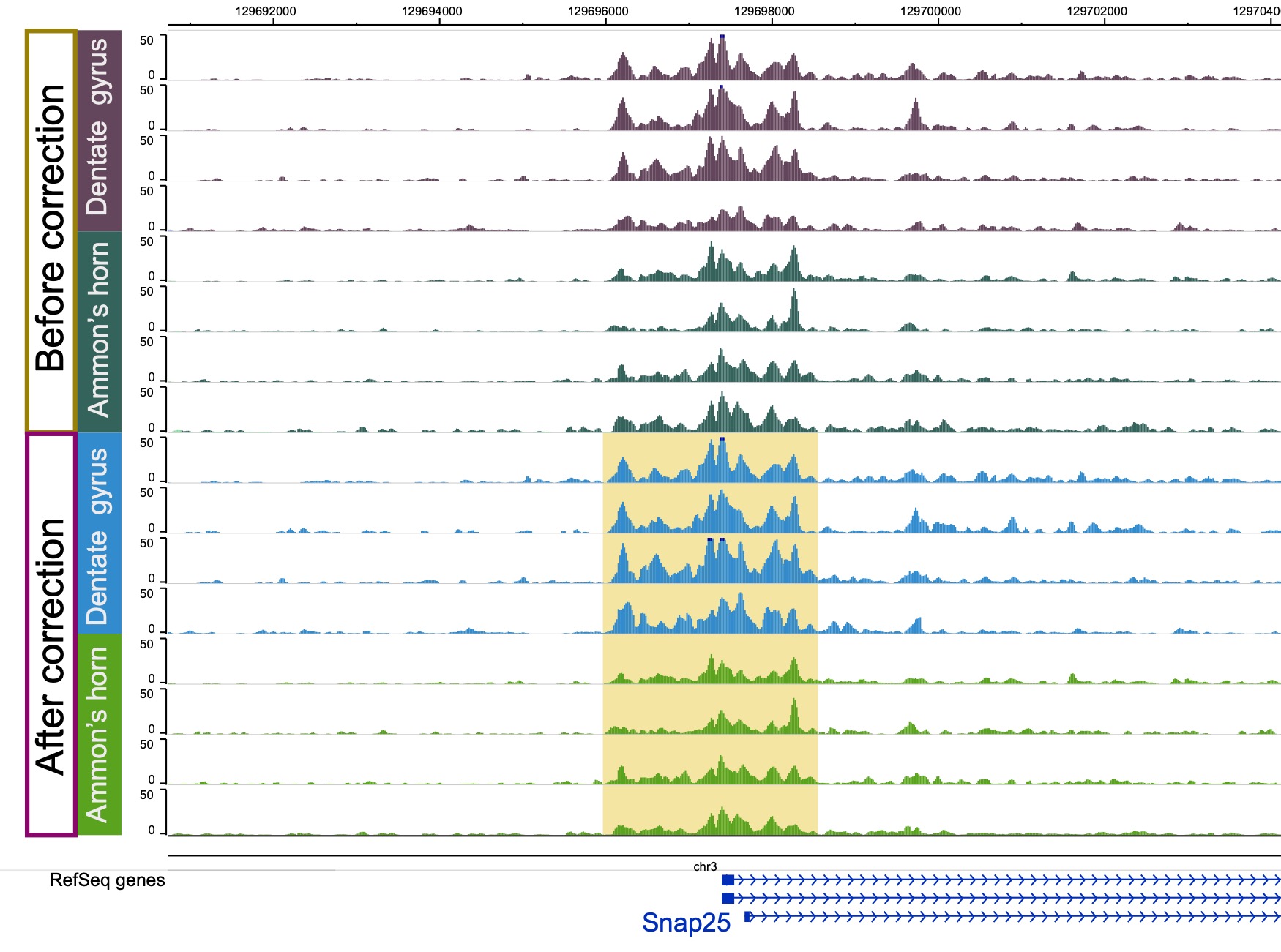


**Supplementary Figure 5. Differential accessible region between Dentate gyrus and Ammon’s horn located in the promoter of Snap25.**

**Supplementary Figure 6: BeCorrect adjusts the bedgraph densities based on four different criteria.**

**Supplementary Figure 7: BeCorrect corrected the technical batch effect (sequencing-depth).**
